# Pulmonary Hypertension Related to Left-Sided Cardiac Pathology

**DOI:** 10.1155/2011/381787

**Published:** 2011-05-29

**Authors:** Todd L. Kiefer, Thomas M. Bashore

**Affiliations:** Division of Cardiology, Duke University Medical Center, P.O. Box 3102, Durham, NC 27710, USA

## Abstract

Pulmonary hypertension (PH) is the end result of a variety of diverse pathologic processes. The chronic elevation in pulmonary artery pressure often leads to right ventricular pressure overload and subsequent right ventricular failure. In patients with left-sided cardiac disease, PH is quite common and associated with increased morbidity and mortality. This article will review the literature as it pertains to the epidemiology, pathogenesis, and diagnosis of PH related to aortic valve disease, mitral valve disease, left ventricular systolic and diastolic dysfunction, and pulmonary veno-occlusive disease. Moreover, therapeutic strategies, which focus on treating the underlying cardiac pathology will be discussed.

## 1. Introduction

Pulmonary hypertension (PH) occurs with an overall prevalence estimated at 15 per one million individuals [1]. It is the end result of a variety of disparate pathophysiologic processes. Ultimately, these disease states lead to a spectrum of histopathologic lesions in the pulmonary vasculature with differing degrees of hypertrophy of the medial layer of the vessel wall, hyperplasia of the intimal layer, proliferation of the adventitial layer, and/or plexiform lesions [1]. These changes in the structure of the pulmonary arterial vascular bed lead to resistance to blood flow, and correspondingly, increased right ventricular (RV) pressures often leading to RV pressure overload with eventual RV failure.

**Figure 1:**
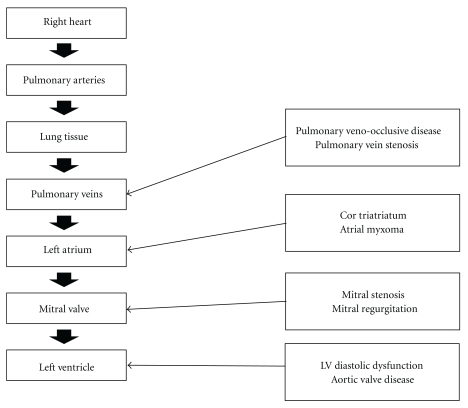
Anatomic organization of left heart causes of pulmonary hypertension from the right ventricle through the lungs to the left ventricular outflow tract.

The most up-to-date classification system categorizing patients with PH into groups based on the underlying disease process leading to PH was published in the European Society of Cardiology Guidelines in 2009 (Table 1) [2]. Of these groups, patients with Group 1 PH, not including pulmonary venoocclusive disease (PVOD), have been most extensively studied in pharmacotherapy clinical trials. In addition, PH is very common in patients with left-sided cardiac disease and has been reported in greater than 60% of patients with left ventricular systolic dysfunction, greater than 80% of patients with left ventricular diastolic dysfunction, and in 78% of patients prior to mitral valve surgery [3–6]. This article will review the current literature pertaining to PH secondary to left-sided cardiac disease (Group 2 PH), including PVOD (classified as Group 1′ PH), pulmonary vein stenosis, mitral stenosis (MS), mitral regurgitation (MR), aortic stenosis (AS), aortic regurgitation (AR), and left ventricular systolic/diastolic dysfunction (Figure 1). In addition, it is worth mentioning that a web membrane, between the pulmonary veins and the left atrial chamber, (cor triatriatum sinister) and left ventricular inflow obstruction from a left atrial myxoma have also been associated with pulmonary hypertension. Other lesions that place a pressure overload on the left ventricle (LV), such as systemic hypertension, and the rare clinical entities of coarctation of the aorta, supravalvular aortic stenosis, and a subaortic membrane, have been reported in association with PH. However, they will not be discussed in detail individually.

## 2. Diagnosis

Consensus guidelines define pulmonary arterial hypertension (PAH) as a mean pulmonary artery (PA) pressure greater than 25 mm Hg in the setting of a pulmonary capillary wedge pressure (PCWP), left atrial (LA) pressure, or left ventricular end-diastolic pressure (LVEDP) less than or equal to 15 mm Hg [2]. Meanwhile, the term PH, in a less specific manner, refers to a mean PA pressure >25 mm Hg due to any cause [2]. 

Transthoracic echocardiography is recommended as a screening test in the evaluation of suspected PAH, and this will provide essential information regarding concomitant left-sided valvular or ventricular dysfunction [1]. In some instances, however, invasive hemodynamic evaluation with right heart catheterization is required to confirm the diagnosis as echocardiography often underestimates the PA pressures and does not provide an accurate assessment of PCWP [1].

Careful analysis of the invasive hemodynamic data is critical to making a correct diagnosis and recommending the appropriate therapeutic options. As will be discussed in detail in following sections, the vast majority of patients with PH in the setting of an elevated PCWP should not be treated with PAH vasodilator therapies. In order to ensure the accuracy of the PCWP data, close attention should be given to the fidelity of the a-, c-, and v-wave morphologies on the PCWP hemodynamic tracing. Furthermore, the PCWP should be measured at end-expiration (intrapleural pressure is about −5 mm Hg at that point). If there is any question about the PCWP validity, then some advocate a PCWP wedge saturation (it should be similar to pulmonary venous saturation if done properly) and/or direct measurement of the LVEDP for confirmation.

Vasodilator challenge is an integral element in the assessment of suspected PAH and should be conducted for patients with a mean PA pressure ≥25 mm Hg and a PCWP ≤15 mm Hg. For patients with a PCWP >15 mm Hg, vasodilator testing should generally not be performed, or if it is performed, then it should be done with close hemodynamic monitoring by experienced clinicians with expertise in the evaluation of PAH due to the risk for development of acute pulmonary edema and sudden respiratory compromise. Approximately 10% of patients with PAH have a positive response to vasodilator challenge, defined as a decrease in mean PA pressure by >10 mm Hg to an absolute mean <40 mm Hg without a decrease in systemic blood pressure or cardiac output [2]. Inhaled nitric oxide, which has a very short half-life (15–30 seconds), is most frequently used to assess vasoreactivity [2, 7, 8]. Other agents including intravenous nitroprusside, epoprostenol, and adenosine have also been used for vasodilator response testing [2]. For patients with a cardiac shunt, nitroprusside should generally be avoided, as this agent is also a potent arterial vasodilator that will decrease systemic vascular resistance (SVR) and may facilitate right to left shunting leading to increasing hypoxemia.

## 3. Obstruction to Pulmonary Venous Drainage

Pulmonary venoocclusive disease (PVOD) is a rare entity characterized by obliterative fibrosis of small pulmonary veins [9]. It is estimated that PVOD occurs with a prevalence of 0.1-0.2 per one million in the population and is the cause of 10% of the cases of PAH [9]. Significant morbidity and mortality is observed from PVOD-associated PAH with a one-year mortality of 72% [10]. Its cause is unknown but has been associated with viral infections and as a complication of certain diseases such as scleroderma, systemic lupus erythematosus, leukemia, chemotherapy, or bone marrow transplantation. 

**Figure 2:**
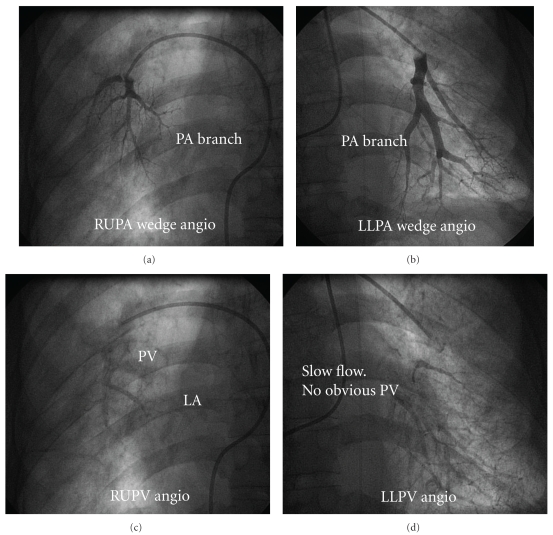
Pulmonary vein wedge angiography. In panel (a) balloon occlusion with hand contrast injection demonstrates opacification of the distal branches of the right upper pulmonary artery (RUPA) and in panel (b) the levophase of the wedge angiogram shows normal right upper pulmonary vein (RUPV) drainage into the left atrium (LA). Panel (c) shows normal opacification of the distal branches of the left lower pulmonary artery (LLPA) with wedge angiography. However, during the levophase in panel (d), there is abnormal drainage of the left lower pulmonary vein (LLPV) with contrast persisting in the LLPA and the absence of contrast media in the LA diagnostic of PVOD.

Given the relative infrequency of PVOD in the population, it is a challenging disease to characterize and study. Small studies have suggested a statistical association between PVOD among male gender and a history of smoking when compared to a population of idiopathic PAH patients [11]. The clinical presentation of PVOD varies but often includes dyspnea with exertion, hemoptysis, pulmonary edema, and PAH. One clue is often the presence of segmental pulmonary edema—something that should not occur with idiopathic PAH. Otherwise, differentiating PAH due to PVOD from other causes based on clinical and noninvasive evaluation can be challenging. One study reported statistically lower PaO2 and DLCO values in PVOD compared with idiopathic PAH patients [11]. However, these are not specific findings unique to PVOD. In the past, lung ventilation-perfusion scanning was part of the diagnostic algorithm for the evaluation of suspected PVOD, but this is no longer recommended due to nonspecific findings and overlap with patterns of chronic thromboembolic disease [9]. Right heart catheterization demonstrates PAH but often with a lower mean PA systolic and mean RA pressure than in patients with idiopathic PAH [11]. The PCWP is often normal with a blunted waveform but should be carefully measured in multiple, bilateral lung zones as a notable difference in PCWP in one lung zone suggests PVOD. Thus, if PVOD is suspected, it is crucial at right heart catheterization that the PCWP be measured in both upper and lower lung fields. Wedge angiography can confirm the diagnosis (Figure 2). The wedge angiogram is performed using a balloon-tipped catheter and hand-injecting contrast media to fill the distal pulmonary arterial bed. Release of the balloon allows noncontrasted blood to wash out and opacify the pulmonary venous system. By assessing the pulmonary venous system in each quadrant, failure to visualize the respective pulmonary vein in a quadrant is diagnostic of the disease. Additionally, when PVOD is considered the likely diagnosis, then vasodilator challenge with inhaled nitric oxide should be avoided, or undertaken with caution and low-dose administration, given multiple reports of severe pulmonary edema developing after vasodilator challenge in PVOD patients [9]. Thus, the diagnosis of PVOD is made from a combination of the above characteristics in the absence of left ventricular diastolic dysfunction, left-sided valve dysfunction, or any other identifiable cause for PH.

Despite advances in the management of patients with idiopathic PAH using vasodilator therapy, clinical improvement has not been seen in PVOD-related PAH, and many patients actually clinically deteriorate with the onset of severe pulmonary edema after initiation of such agents due to the obstruction in pulmonary venous outflow. Several recent reports have suggested that epoprostenol may play a role in bridging patients with PVOD to lung transplantation [9]. However, lung or heart-lung transplantation remains the only effective therapy, and patients should be referred to a transplantation center upon diagnosis.

Finally, other obstructive lesions to pulmonary venous drainage have been observed clinically and reported in the literature infrequently. Pulmonary vein stenosis is one of these lesions and can be either congenital or acquired. Acquired pulmonary vein stenosis has been noted after electrophysiologic ablation procedures and in patients with sclerosing mediastinitis. Also, cor triatriatum sinister and atrial myxoma have the potential to impair pulmonary venous drainage into the left atrium with the subsequent development of PH.

## 4. Mitral Stenosis and Regurgitation

Elevation of PA pressures is commonly observed in patients with both MS and MR. The presence or absence of PH with mitral valve disease is a key element in the decision-making algorithm for percutaneous or surgical intervention on the mitral valve in the most recent American College of Cardiology/American Heart Association Valvular Heart Disease guidelines [12]. 

The mechanism by which PH develops in patients with mitral valve disease is driven by an elevation of LA pressure, which in turn, leads to pulmonary venous hypertension, and subsequently, pulmonary arterial hypertension to deliver deoxygenated blood across the lungs from the right heart to the left heart. Left atrial compliance is often reduced resulting in an increased “v” wave in mitral valve disease. In patients with MS, the elevation in LA pressure also results from the abnormal diastolic pressure gradient across the dysfunctional mitral valve, while in patients with chronic MR, a further increase in LA pressure results from the regurgitant systolic jet and the rise in LV end-diastolic pressure.

With MS, there are two well-characterized associated hemodynamic states. Initially, with progression of MS, the mitral valve area (MVA) and cardiac output decrease with a concomitant rise in the LA pressure [13]. Later in disease progression, with a continued reduction in MVA and elevation of LA pressures, changes in the pulmonary vascular bed ensue, increasing the pulmonary vascular resistance and PH. Often overt right heart failure occurs from right ventricular (RV) pressure overload [13]. Thus, the early stages of moderate to severe MS are associated with a decline in cardiac output due to one lesion at the level of the mitral valve. However, as the obstruction to LV filling by the stenotic mitral valve worsens with the onset of PH, this “secondary stenosis” at the pulmonary bed level may lead to both low systemic cardiac output and RV failure.

Mitral stenosis, when moderate to severe, is associated with a variable degree of PH in the majority of patients. The pathophysiology of PH in MS involves structural alteration of the pulmonary vascular bed mediated by the potent vasoconstrictor endothelin-1 (ET-1) [14]. Levels of ET-1 are three-fold higher in patients with severe MS compared with healthy control subjects [14]. In a group of patients with severe MS undergoing PBMV or mitral valve replacement (MVR), the baseline ET-1 concentration is an independent predictor of a decrease in PCWP at 6 months following mitral valve intervention [14].

The American College of Cardiology (ACC)/American Heart Association (AHA) Valvular Heart Disease guidelines recommend intervention to relieve MS with percutaneous balloon mitral valvuloplasty (PBMV) if the valve morphology is favorable with a Wilkins score less than 8 and less than moderate MR, or surgical MVR, when PH is present [12]. More specifically, PBMV is advised for asymptomatic patients with moderate or severe MS and a resting PA systolic pressure greater than 50 mm Hg or a PA systolic pressure greater than 60 mm Hg and/or a PCWP greater than 25 mm Hg with exercise [12]. For patients with NYHA class II-IV symptom status and moderate to severe MS, PBMV is recommended if the criteria above are met [12]. If valve morphology is not amenable to PBMV or there is also moderate to severe MR, then surgical valve replacement should be pursued even if severe PH is present [12].

A large body of outcome data exists for PBMV in mitral stenosis. Early work in the PBMV era showed that PA pressures fell immediately after PBMV, in concert with the reduction in mitral valve gradient [15]. In the immediate post-procedure period, PVR declined in this study population from 630 to 447 dynes × sec/cm^5^ and subsequently exhibited additional reduction to 280 dynes × sec/cm^5^ at 7-month follow-up catheterization [15, 16]. Further work in this area confirmed a reduction in PA pressures and PVR with PBMV [17, 18]. Thus, one would expect relief of the MS by PBMV to reduce PA pressures to normal or near-normal levels in most patients. Following PBMV, surveillance for mitral valve restenosis with the return of PH is necessary. Appropriately selected patients with PH and MS should be referred to tertiary care centers with interventional experience in PBMV. 

Surgical correction with mitral valve repair or replacement is the treatment of choice for chronic severe mitral regurgitation. For patients with increased predicted perioperative morbidity and mortality, percutaneous intervention with the MitraClip device may be considered. The most recent ACC/AHA management guidelines endorse mitral valve intervention if symptoms are present or in asymptomatic patients with a left ventricular ejection fraction (LVEF) less than 60%, a left ventricular end-systolic diameter greater than 4 cm, a resting PA systolic pressure greater than 50 mm Hg, or a PA systolic pressure greater than 60 mm Hg with exercise [12]. 

Most of the clinical studies evaluating surgery for patients with mitral valve disease and PH included both MS and MR. Thus, the surgical outcomes for MS and MR will be reviewed together. Without relief of mitral valve obstruction, observational data from several decades ago demonstrated that mean survival was 2.4 years when severe PH was present with MS [19]. Several small, observational studies initially reported over forty years ago examined the changes in PA pressures pre- and postoperatively with right heart catheterization in patients with PH and mitral valve disease. One study detailed the hemodynamic findings in 31 patients before and at a mean of 7 months after Starr-Edwards prosthetic MVR [20]. In this study, PA systolic pressures declined from 75 mm Hg to 39 mm Hg with a concomitant increase in the cardiac index from 2.04 to 2.99 liters/minute/meter^2^ [20]. There was no difference in hemodynamic response to surgery between those with MS and MR. Similar work from the same era documented hemodynamic evaluation from a preoperative baseline over the course of 8-9 days postoperatively [21]. The mean PA pressures decreased from 71 mm Hg to 35 mm Hg with an increase in the cardiac index from 1.7 to 4.0 liters/minute/meter^2^ from baseline values to the end of the study period [21]. Another study from the same time period evaluated 25 patients at rest and with exercise to right heart catheterization preoperatively and at one year following Starr-Edwards prosthetic MVR [22]. This work reported a decline in PA pressures in 68% of patients and an improvement in cardiac index in the majority of patients one year after mitral valve surgery [22]. All patients in the series demonstrated persistent exercise-induced PH, which in most, was related to increased gradients across the mitral valve during exercise [22]. 

Approximately 25 years after this original work, invasive hemodynamic evaluation from 22 patients with rheumatic mitral valve disease at preoperative baseline, and at 6 and 12 months following postoperative baseline was published [23]. This study demonstrated a significant diminution of PA systolic pressures and PCWP at rest and with exercise from baseline preoperative values to 6 months following surgery [23]. Furthermore, from 6 to 12 months postoperatively, there was an additional decrease in PA systolic pressures during exercise [23]. 

 Finally, the acute hemodynamic response to bileaflet mechanical MVR was reported in 60 patients with PH from the modern era [24]. This publication evaluated 30 patients with mild PH (mean PA pressure of 29 mm Hg) and 30 patients with severe PH (mean PA pressure of 54 mm Hg) using invasive hemodynamic monitoring for 48 hours postoperatively [24]. In the cohort with mild PH, the mean PA pressure fell to 16 mm Hg, while the cohort with severe PH had a mean PA pressure of 23 mm Hg at 48 hours postoperatively [24]. Over 40 years of hemodynamic research suggests that most patients have an improvement in PA pressures to near-normal values following mitral valve surgery with some patients exhibiting normal resting pressures and persistent exercise-induced PH. 

Patients with PH secondary to mitral valve disease have been excluded from the clinical trials evaluating the current United States Food and Drug Administration (FDA)-approved PAH pharmacologic vasodilator therapies. Case reports describing the safety and efficacy of various vasodilator agents during the immediate postoperative period in patients undergoing mitral valve surgery with preoperative PH have been published. The agents utilized include inhaled prostacyclin, inhaled nitric oxide, intravenous nitroprusside, and inhaled iloprost, and the reports focused on acute hemodynamic changes with the respective agents [25–29]. The pharmacologic mechanisms of these agents target different cellular pathways. Inhaled iloprost and epoprostenol are prostacyclin derivatives, which stimulate cyclic adenosine monophosphate (cAMP) production leading to pulmonary arterial smooth muscle relaxation [30]. In a similar manner, milrinone, a type 3 cAMP phosphodiesterase inhibitor, produces systemic and pulmonary arterial vasodilation by blocking the metabolism of cAMP in smooth muscle cells [31]. Through a different pathway with stimulation of cyclic guanylate monophosphate synthesis, inhaled nitric oxide and nitroprusside influence pulmonary arterial smooth muscle relaxation and vasodilation [30]. However, all of the agents are associated with a decrease in mean PA pressure, PVR, and with an increase in cardiac output. Moreover, one publication reports a greater likelihood for separation from cardiopulmonary bypass after mitral valve surgery in patients with PH treated with inhaled iloprost compared with no vasodilator therapy [29]. Pulmonary vasodilator agents appear safe for short-term administration in patients with PH during the perioperative mitral valve surgery period without any adverse events reported. Careful hemodynamic monitoring of the PCWP is necessary during administration to avoid the potential development of decompensated HF and pulmonary edema.

In addition, there are isolated case reports detailing the use of PAH pharmacotherapies as chronic therapy in patients with mitral valve disease and PH. The use of epoprostenol in this manner was reported in a patient with residual severe PH after MVR. There was an improvement in functional status and hemodynamics with a decrease in mean PA pressure and an increase in cardiac output [32]. Likewise, the successful use of the oral pulmonary vasodilator, sildenafil, following MVR in a patient with persistent severe PH has been noted [33]. The future role, if any, that conventional PAH drug therapy may play in the management of patients with PH and mitral valve disease that persists after surgery, or in patients who are not candidates for surgical or percutaneous intervention, remains to be determined through rigorous scientific evaluation.

## 5. Aortic Stenosis

Valvular AS is one of the most frequently encountered pathologies in the practice of adult cardiovascular medicine. However, the association of PH with severe AS is often underappreciated and varies with the threshold used for the detection of the presence of PH. Aortic stenosis results in PH by creating LV diastolic dysfunction and subsequent pulmonary venous hypertension due to associated LV hypertrophy and reduced LV diastolic function. One of the original systematic characterizations of PH in patients with severe AS reported a prevalence for PH of 50% using the threshold of a PA systolic pressure of >30 mm Hg [34]. In this publication, PH was statistically associated with an elevated LVEDP [34]. Subsequently, using a cutoff value of a PA systolic pressure >50 mm Hg, 29% of patients with severe AS had concomitant PH [35]. More recently, in a cohort of nearly 400 patients with symptomatic severe AS, 50% had mild to moderate PH with mean PA pressures of 31–50 mm Hg and 15% had severe PH with a mean PA pressure >50 mm Hg [36]. Of note, in both of these studies, the majority of patients had an elevated transpulmonary gradient (TPG) suggesting that over time changes in the pulmonary vasculature had occurred leading to PH out-of-proportion to the PCWP/LVEDP.

Several echocardiographic studies have examined the relationship between severe AS and PH. In a small study involving 50 patients with severe AS and PH, multivariate analysis revealed that diastolic function as assessed by E/e' was the only independent predictor of PH [37]. However, in a larger study involving 626 patients, multivariate analysis showed that lower LVEF, severity of concomitant mitral regurgitation, smaller aortic valve area, and not taking a statin medication independently predicted PH [38]. These data suggest that additional left-sided cardiac pathology in addition to diastolic filling abnormalities may increase the likelihood for developing PH in patients with severe AS. Thus, careful hemodynamic evaluation is required to detect the presence of PH and any associated lesions given the increased surgical morbidity and mortality with aortic valve replacement when PH is present.

Surgical aortic valve replacement (AVR) is the recommended therapeutic intervention for patients with symptomatic, severe AS with a mean gradient greater than 40 mm Hg or an aortic valve area less than 1.0 cm^2^ [12]. Likewise, AVR is advised in the asymptomatic patient when the LVEF is less than 50% [12]. However, perioperative morbidity and mortality increase significantly when PH is present. This is partly due to persistent pulmonary hypertension immediately after AVR, since LV diastolic dysfunction improves only after there is LV remodeling following AVR, and this may take several months.

 In one study, the characteristics of 47 patients with severe AS and severe PH during the time period of 1987 to 1999 were analyzed, and the outcome demonstrated that perioperative mortality was 16% [39]. For the group of patients who had valve surgery and survived, PA pressures gradually declined, with an improvement in New York Heart Association (NYHA) class and LVEF [39]. The benefit from AVR, though, can be striking; a retrospective analysis of a cohort of 116 patients with severe AS and severe PH showed a 30-day mortality of 8% in patients who had AVR versus 30% for those not having AVR [40]. This statistically significant survival difference persisted with 34% mortality in those who underwent AVR and 80% mortality in those without valve replacement at 5 year followup [40]. In addition, AVR was associated with a survival benefit after multivariate logistic regression analysis to control for other variables of comorbidity and with the use of a propensity score adjustment [40]. 

Based on analysis of nonrandomized, observational data, AVR in patients with severe PH and AS is associated with increased perioperative mortality compared to patients without PH. However, AVR is also associated with improved long-term survival and should be considered in selected patients at experienced, high-volume surgical centers. Based on the dramatic results of initial clinical trial data, transcatheter aortic valve replacement will also likely be an option in the future for patients with severe AS and PH who are at high risk for surgical AVR due to other comorbid conditions. At the present time, even isolated case reports on the use of pulmonary vasodilators in patients with severe PH and AS are lacking from the literature and the use of such agents cannot be recommended.

## 6. Aortic Regurgitation

Elevation of pulmonary artery pressures secondary to isolated aortic valve regurgitation (AR) is less common than with other valve lesions but does occur. Prior studies have reported a prevalence of PH in 10–20% of patients with severe AR [41]. The pathophysiology is explained by a chronic elevation of the LVEDP, which in turn, leads to an increase in LA and PA pressures or due to the acute elevation in LVEDP with acute severe AR (such as what might be observed in endocarditis or aortic dissection).

In chronic AR, surgery to replace the aortic valve is indicated when there are symptoms present with severe AR or with asymptomatic severe AR when the LVEF is less than 50% or there is dilation of the LV [12]. Retrospective analysis of 139 patients with PH and AR was reported nearly twenty years ago. This work observed that there was no significant difference in operative mortality or postoperative complications in patients undergoing AVR with severe PH and severe AR compared with mild or no PH and severe AR [42]. Furthermore, PA pressures declined to near-normal values in the vast majority of patients following AVR [42]. More recently, a single-center retrospective study of 506 patients with severe AR demonstrated that severe PH was statistically associated with lower LVEF, greater LV end-diastolic and end-systolic dimensions, and a higher grade of concomitant MR [41]. Moreover, multivariate analysis with propensity score adjustment showed an independent association between AVR and survival in patients with both severe PH and severe AR during 5 years of followup [41]. 

Although limited by potential selection bias, this work suggests that AVR can be performed with acceptable perioperative risk in patients with severe PH due to AR. In addition, it also highlights the recurrent theme that valve surgery is often associated with a significant improvement in PA pressures and improved survival based on observational datasets.

## 7. Left Ventricular Diastolic Dysfunction Associated with Preserved Systolic Left Ventricular Function

Heart failure with preserved systolic function accounts for over half of hospitalizations for congestive heart failure. This category represents a varied group of disease states including systemic hypertension, hypertrophic cardiomyopathy, infiltrative cardiomyopathies, Fabry's disease, and obstructive sleep apnea. Some of these patients will develop PH as a response to the abnormal diastolic filling of the LV. Likewise, there is a growing population of elderly patients with dyspnea who have PH in which HF with preserved LVEF appears to be the most common cause [43]. The common link between all of these pathologies is the impairment of diastolic filling. Over time this leads to an increase in LA pressure in order to adequately fill the LV during diastole and a reduction in LA compliance. Subsequently, with the increase in LA pressure, there is a corresponding rise in PV and PA pressure. In some patients with long-standing elevation of LA pressure, the TPG gradient rises out of proportion to the LA pressure. 

The epidemiology and association of PH in patients with normal LV function and diastolic dysfunction have been well recognized over the last decade. A recent population-based study of 244 patients with HF and preserved LVEF observed PH in 83% of patients as defined by an echocardiographic Doppler estimation of PA systolic pressure greater than 35 mm Hg [5]. Furthermore, PH in patients with HF and preserved LV function has been found to be a strong predictor of mortality during a 2.8-year follow-up period [5]. This will likely be an increasing clinical problem in the coming years with the aging population and the epidemic of diabetes mellitus and obesity. 

For the diagnosis of PH related to impaired diastolic filling, other potential causes of PH must be excluded. Right heart catheterization is obligatory and will usually reveal an elevated PCWP and LVEDP, mean PA pressure, and in some patients an elevated PVR with an exaggerated TPG gradient. Although not part of the diagnostic criteria in the PH guidelines, invasive hemodynamic evaluation with supine bicycle or arm weight exercises may occasionally be useful to better understand the symptomatic limitation of individual patients with suspected PH due to diastolic dysfunction [44].

At the present time, there are no guideline recommendations or clinical trial data regarding the management of PH in diastolic HF [1]. General guidance on the management of HF with preserved LV function has been published, however, emphasizing the importance of control of systemic blood pressure, rate control for atrial fibrillation if present, and diuretic usage if needed to avoid hypervolemia [45]. In the future, results from the currently enrolling Evaluating the Effectiveness of Sildenafil at Improving Health Outcomes and Exercise Ability in People with Diastolic Heart Failure (RELAX) trial may provide information on the use of sildenafil pulmonary vasodilator therapy in this specific patient population [46].

## 8. Left Ventricular Diastolic Dysfunction Associated with Left Ventricular Systolic Dysfunction

Pulmonary hypertension is commonly found in patients with left ventricular systolic dysfunction. It has been reported that two-thirds to three-fourths of patients with systolic heart failure (HF) due to ischemic or nonischemic cardiomyopathy have associated PH [31]. However, the presence or severity of PH does not correlate with LVEF [47]. The greatest predictors of PH in a population with LV systolic dysfunction are the grade of MR and mitral inflow E-wave deceleration time [48]. The latter reflects the rapid rise of LV diastolic pressure and decline in filling when there is diastolic dysfunction. Hence, the degree of LV systolic dysfunction is not the primary characteristic responsible for the development of PH, but rather the degree of LV diastolic filling impairment and associated functional MR.

Greater understanding of the physiological mechanisms of PH in HF with systolic dysfunction has evolved over the last two decades. The circulating peptide ET-1 is a potent vasoconstrictor and seems to play a role in the development of PH with MR. Elevated levels of circulating ET-1 in HF have been linked to higher PA pressures and PVR [49]. Moreover, ET-1 concentration has a strong positive correlation with NYHA class and a strong inverse relationship with LVEF and cardiac index [50]. Thus, the ET-1 receptor represents a logical therapeutic target.

Symptoms such as shortness of breath at rest and with exertion are a major manifestation of systolic HF, which negatively impact activity level and quality of life. In patients with a reduced LVEF, the concomitant presence of PH correlates with more advanced symptom status and greater functional impairment as reflected by a statistically higher NYHA class than a similar cohort with LV systolic dysfunction without PH [3]. This effect has been objectively documented with cardiopulmonary exercise (CPX) testing. CPX testing in 320 patients with an LVEF less than 40% demonstrated that cardiac output and peak oxygen consumption with exercise were significantly lower in those with an elevated PVR, further emphasizing the association of PH on symptom status and hemodynamics. 

When PH is present with systolic HF, it is also associated with increased risk of death [51, 52]. One study which followed 400 patients for 5 years estimated that there was a 9% increase in mortality for every 5 mm Hg increase in right ventricular systolic pressure using Cox proportional hazards statistical analysis to adjust for other variable known to impact mortality [53]. Given the high mortality for patients with HF due to LV systolic dysfunction at 5 years, the development of PH, which appears to further increase the risk of death, represents a serious problem.

Selected patients with advanced HF symptoms and severe LV systolic dysfunction are often considered for orthotopic heart transplantation. Multiple studies have examined the impact of PH on outcomes in patients undergoing transplant. The synthesis of the various studies shows that when a PVR greater than 2.5 Wood units and a TPG gradient greater than 15 mm Hg is present, there is an increase in mortality at 3 month and 1 year posttransplant [47]. Mortality at one year posttransplant was 5.6% with a PVR less than 2.5 Wood units and a TPG gradient less than 15 mm Hg, while it was 24.4% in those with hemodynamics exceeding these threshold values [54]. Thus, PH with a PVR greater than 5 Wood units is a relative contraindication to transplant based on the International Society for Heart and Lung Transplantation guidelines [55]. Moreover, vasodilator challenge should be assessed during right heart catheterization to evaluate whether the elevated PVR is fixed or vasoreactive [55]. Observational data has shown that there is a significant reduction in mortality at 3 month posttransplant in patients with a pretransplant PVR >2.5 Wood units in whom the PVR decreased <2.5 Wood units with nitroprusside administration when compared to patients without such a decline in PVR (3.8% versus 40.6%) [56]. In a recent small pilot study, 6 pretransplant patients with a TPG gradient greater than 12 mm Hg, a PVR greater than 2.5 Wood units, and no reversibility with intravenous (IV) nitroprusside were given sildenafil for one month [57]. After one month of treatment, three patients demonstrated a normalization of TPG and PVR and 2 patients exhibited a decline in PA pressures with IV nitroprusside [57]. It is anticipated that future inquiry will expand our understanding of the use of pulmonary vasodilator therapies in the pretransplant population. Moreover, resolution or improvement in PH has also been reported in patients who have had recovery of LV function or who have undergone cardiac transplantation or left ventricular assist device (LVAD) placement. 

There appears to be an expanding role for the LVAD as a bridge to heart transplant in the management of patients with severe left ventricular systolic HF and PH. Several studies in patients with systolic HF and severe PH have demonstrated that an elevated PVR despite pharmacologic therapy is often reduced to <2.5 Wood units over a 6 month time period following LVAD placement [58–62]. Retrospective, observational data analysis has also established that in patients in whom the pulmonary pressure improves with LVAD therapy as bridge to transplant, there is no statistical difference in subsequent posttransplant survival compared to patients without PH [60, 61]. The beneficial actions of the LVAD undoubtedly relate to afterload reduction of the LV and a reduction in the PCWP with corresponding reductions in the mean PA pressure and PVR. Improvement in the PVR has been reported with both pulsatile flow devices and newer, continuous flow LVADs [63]. Given the strong correlation of an elevated PVR and an adverse outcome following orthotopic heart transplant, the use of LVAD therapy to reduce the PVR prior to transplant may improve the transplant outcomes in these patients.

Although there are no FDA-approved agents for the treatment of PH due to LV systolic dysfunction, the use of long-term pulmonary vasodilator therapy has been attempted to improve symptoms. Early work in this area with chronic epoprostenol administration in the Flolan International Randomized Survival trial (FIRST), however, observed an increased mortality in the cohort with LV systolic dysfunction; the trial was terminated early by the Data Safety Monitoring board [64]. Subsequently, small series have investigated the efficacy and hemodynamic actions of other vasodilator therapies in patients with systolic HF and PH. As described earlier, endothelin-1 likely plays an important role in the pathophysiology of PH in this setting. Therefore, drugs targeting inhibition of the endothelin receptor have been developed and are approved for chronic therapy in idiopathic PAH. Unfortunately, long-term studies involving the endothelin receptor antagonists, darusentan and bosentan, have not shown any beneficial actions on LV chamber size, or neurohormonal levels with darusentan, or on symptoms with bosentan [65, 66]. Another agent, nesiritide, a recombinant version of human brain natriuretic peptide, has also been extensively studied with mixed results. One of the hemodynamic actions of nesiritide is to decrease the PCWP, the mean PA pressure, and PVR acutely with short-term infusion [67].

Milrinone, an inhibitor of type 3 phosphodiesterase, is an inotrope and systemic vasodilator used in the management of acute decompensated HF from LV systolic dysfunction. It also has hemodynamic actions on the pulmonary vasculature by decreasing PVR and augmenting RV function [68]. In clinical practice, it is used in selected patients as a bridge to transplant or in the perioperative period after LVAD placement in patients with systolic HF and PH.

 Finally, the type 5 phosphodiesterase inhibitor, sildenafil, which is approved for chronic therapy of idiopathic PAH, has been evaluated in PH with systolic HF. During a 6 month randomized trial of sildenafil or placebo in 46 patients with mild PH and systolic HF, there was statistically significant reduction in PA pressures and an increase in peak oxygen consumption during CPX testing, without adverse side effects reported [69]. Similarly, the randomization of 34 patients to sildenafil or placebo showed an improvement in PVR, peak oxygen consumption during CPX testing, 6-minute walk duration, and quality of life with sildenafil therapy at 3 months [70]. Larger scale clinical trials with longer followup are needed, though, to determine what role, if any, sildenafil will play in the management of PH in systolic HF. The cornerstone of therapy for patients with PH and LV systolic dysfunction remains evidence-based HF therapies such as beta-blockers, angiotensin-converting enzyme inhibitors, and aldosterone antagonists, which reduce afterload on the LV and influence favorable myocardial remodeling leading to improved LV diastolic properties, and in turn, lower LA pressure with a corresponding reduction in PA pressures.

## 9. Conclusion

In conclusion, PH frequently develops in response to left-sided cardiac disease due to elevated pulmonary venous pressure and is associated with a series of lesions that range from diseases of the pulmonary veins, mechanical obstruction in the LA or at the mitral valve, or to elevation in the left atrial and pulmonary venous pressure due to mitral regurgitation, abnormal LA compliance, or to LV diastolic dysfunction. In some patients, secondary pulmonary hypertension appears to occur in addition. Pulmonary hypertension is associated with increased morbidity and mortality. In the setting of PH-related to left-sided obstructive or regurgitant valve disease, the PA pressures commonly decrease significantly or return to normal after valve replacement, repair, or mitral valvuloplasty; unfortunately this does not occur in all. 

 For patients with left ventricular systolic/diastolic dysfunction, the prognosis and options for treatment of associated pulmonary hypertension independent of therapy for the left heart disease are less favorable. Although not included in the vast majority of PAH vasodilator therapy clinical trials, vasodilator therapy may be of clinical benefit in a few carefully selected patients with left-sided cardiac disease in whom PH does not improve after addressing the underlying cardiac pathology.

## Figures and Tables

**Table 1:** Classification system of pulmonary hypertension into groups 1–5 based on underlying disease process.

Group 1	Group 1′	Group 2	Group 3	Group 4	Group 5
(i) Idiopathic (ii) Familial (iii) Connective tissue diseases (iv) Congenital shunt lesions (v) HIV (vi) Drugs/Toxins (vii) Hemoglobinopathies (viii) Portal hypertension (ix) Persistent pulmonary hypertension of the newborn	PVOD	(i) Left ventricular systolic/diastolic dysfunction (ii) Left-sided valvular dysfunction	Chronic lung diseases and/or hypoxemia	Chronic thromboembolic disease	Miscellaneous (i) Sarcoid (ii) Histiocytosis X (iii) Fibrosing mediastinitis (iv) Myeloproliferative disorders (v) Metabolic storage diseases (vi) Thyroid disease

## References

[1] McLaughlin VV, Archer SL, Badesch DB (2009). ACCF/AHA 2009 expert consensus document on pulmonary hypertension. A report of the American College of Cardiology Foundation task force on expert consensus documents and the American Heart Association Developed in Collaboration with the American College of Chest Physicians; American Thoracic Society, Inc.; and the Pulmonary Hypertension Association. *Journal of the American College of Cardiology*.

[2] Gali N, Hoeper MM, Humbert M (2009). Guidelines for the diagnosis and treatment of pulmonary hypertension. *European Heart Journal*.

[3] Ghio S, Gavazzi A, Campana C (2001). Independent and additive prognostic value of right ventricular systolic function and pulmonary artery pressure in patients with chronic heart failure. *Journal of the American College of Cardiology*.

[4] Grigioni F, Potena L, Galiè N (2006). Prognostic implications of serial assessments of pulmonary hypertension in severe chronic heart failure. *Journal of Heart and Lung Transplantation*.

[5] Lam CSP, Roger VL, Rodeheffer RJ, Borlaug BA, Enders FT, Redfield MM (2009). Pulmonary hypertension in heart failure with preserved ejection fraction. A community-based study. *Journal of the American College of Cardiology*.

[6] Walls MC, Cimino N, Bolling SF, Bach DS (2008). Persistent pulmonary hypertension after mitral valve surgery: does surgical procedure affect outcome?. *The Journal of Heart Valve Disease*.

[7] Krasuski RA, Warner JJ, Wang A, Harrison JK, Tapson VF, Bashore TM (2000). Inhaled nitric oxide selectively dilates pulmonary vasculature in adult patients with pulmonary hypertension, irrespective of etiology. *Journal of the American College of Cardiology*.

[8] Krasuski RA, Wang A, Harrison JK, Tapson VF, Bashore TM (2005). The response to inhaled nitric oxide in patients with pulmonary artery hypertension is not masked by baseline vasodilator use. *American Heart Journal*.

[9] Montani D, O’Callaghan DS, Savale L (2010). Pulmonary veno-occlusive disease: recent progress and current challenges. *Respiratory Medicine*.

[10] Palazzini M, Manes A (2009). Pulmonary veno-occlusive disease misdiagnosed as idiopathic pulmonary arterial hypertension. *European Respiratory Review*.

[11] Montani D, Achouh L, Dorfmüller P (2008). Pulmonary veno-occlusive disease: clinical, functional, radiologic, and hemodynamic characteristics and outcome of 24 cases confirmed by histology. *Medicine*.

[12] Bonow RO, Carabello BA, Chatterjee K (2008). 2008 focused update incorporated into the ACC/AHA 2006 guidelines for the management of patients with valvular heart disease. A report of the American College of Cardiology/American Heart Association task force on practice guidelines (Writing Committee to Revise the 1998 Guidelines for the Management of Patients With Valvular Heart Disease). *Journal of the American College of Cardiology*.

[13] Feldman TaG W, Baim DS (2006). Profiles in valvular heart disease. *Grossman’s Cardiac Catheterization, Angiography, and Intervention*.

[14] Snopek G, Pogorzelska H, Rywik TM, Browarek A, Janas J, Korewicki J (2002). Usefulness of endothelin-1 concentration in capillary blood in patients with mitral stenosis as a predictor of regression of pulmonary hypertension after mitral valve replacement or valvuloplasty. *American Journal of Cardiology*.

[15] Levine MJ, Weinstein JS, Diver DJ (1989). Progressive improvement in pulmonary vascular resistance after percutaneous mitral valvuloplasty. *Circulation*.

[16] Ribeiro PA, Zaibag MA, Abdullah M (1993). Pulmonary artery pressure and pulmonary vascular resistance before and after mitral balloon valvotomy in 100 patients with severe mitral valve stenosis. *American Heart Journal*.

[17] Hung JS, Chern MS, Wu JJ (1991). Short- and long-term results of catheter balloon percutaneous transvenous mitral commissurotomy. *American Journal of Cardiology*.

[18] Dev V, Shrivastava S (1991). Time course of changes in pulmonary vascular resistance and the mechanism of regression of pulmonary arterial hypertension after balloon mitral valvuloplasty. *American Journal of Cardiology*.

[19] Ward C, Hancock BW (1975). Extreme pulmonary hypertension caused by mitral valve disease. Natural history and results of surgery. *British Heart Journal*.

[20] Braunwald E, Braunwald NS, Ross J, Morrow AG (1965). Effects of mitral-valve replacement on the pulmonary vascular dynamics of patients with pulmonary hypertension. *The New England Journal of Medicine*.

[21] Dalen JE, Matloff JM, Evans GL (1967). Early reduction of pulmonary vascular resistance after mitral-valve replacement. *The New England Journal of Medicine*.

[22] Morgan JJ (1967). Hemodynamics one year following mitral valve replacement. *The American Journal of Cardiology*.

[23] Zielinski T, Pogorzelska H, Rajecka A, Biedermavn A, Sliwinski M, Korewicki J (1993). Pulmonary hemodynamics at rest and effort, 6 and 12 months after mitral valve replacement: a slow regression of effort pulmonary hypertension. *International Journal of Cardiology*.

[24] Tempe DK, Hasija S, Datt V (2009). Evaluation and comparison of early hemodynamic changes after elective mitral valve replacement in patients with severe and mild pulmonary arterial hypertension. *Journal of Cardiothoracic and Vascular Anesthesia*.

[25] Fattouch K, Sbraga F, Bianco G (2005). Inhaled prostacyclin, nitric oxide, and nitroprusside in pulmonary hypertension after mitral valve replacement. *Journal of Cardiac Surgery*.

[26] Santini F, Casali G, Franchi G (2005). Hemodynamic effects of inhaled nitric oxide and phosphodiesterase inhibitor (dipyridamole) on secondary pulmonary hypertension following heart valve surgery in adults. *International Journal of Cardiology*.

[27] Healy DG, Veerasingam D, McHale J, Luke D (2006). Successful perioperative utilisation on inhaled nitric oxide in mitral valve surgery. *Journal of Cardiovascular Surgery*.

[28] Yurtseven N, Karaca P, Uysal G (2006). A comparison of the acute hemodynamic effects of inhaled nitroglycerin and iloprost in patients with pulmonary hypertension undergoing mitral valve surgery. *Annals of Thoracic and Cardiovascular Surgery*.

[29] Rex S, Schaelte G, Metzelder S (2008). Inhaled iloprost to control pulmonary artery hypertension in patients undergoing mitral valve surgery: a prospective, randomized-controlled trial. *Acta Anaesthesiologica Scandinavica*.

[30] McGoon MD, Kane GC (2009). Pulmonary hypertension: diagnosis and management. *Mayo Clinic Proceedings*.

[31] Shah RV, Semigran MJ (2008). Pulmonary hypertension secondary to left ventricular systolic dysfunction: contemporary diagnosis and management. *Current Heart Failure Reports*.

[32] Elliott CG, Palevsky HI (2004). Treatment with epoprostenol of pulmonary arterial hypertension following mitral valve replacement for mitral stenosis. *Thorax*.

[33] Bomma C, Ventura HO, Daniel G, Patel H (2006). Adjunctive sildenafil for the treatment of pulmonary hypertension after mitral valve replacement. *Congestive Heart Failure*.

[34] Johnson LW, Hapanowicz MB, Buonanno C, Bowser MA, Marvasti MA, Parker FB (1988). Pulmonary hypertension in isolated aortic stenosis. Hemodynamic correlations and follow-up. *Journal of Thoracic and Cardiovascular Surgery*.

[35] Silver K, Aurigemma G, Krendel S, Barry N, Ockene I, Alpert J (1993). Pulmonary artery hypertension in severe aortic stenosis: incidence and mechanism. *American Heart Journal*.

[36] Faggiano P, Antonini-Canterin F, Ribichini F (2000). Pulmonary artery hypertension in adult patients with symptomatic valvular aortic stenosis. *American Journal of Cardiology*.

[37] Casaclang-Verzosa G, Nkomo VT, Sarano ME, Malouf JF, Miller FA, Oh JK (2008). E/Ea is the major determinant of pulmonary artery pressure in moderate to severe aortic stenosis. *Journal of the American Society of Echocardiography*.

[38] Kapoor N, Varadarajan P, Pai RG (2008). Echocardiographic predictors of pulmonary hypertension in patients with severe aortic stenosis. *European Journal of Echocardiography*.

[39] Malouf JF, Enriquez-Sarano M, Pellikka PA (2002). Severe pulmonary hypertension in patients with severe aortic valve stenosis: clinical profile and prognostic implications. *Journal of the American College of Cardiology*.

[40] Pai RG, Varadarajan P, Kapoor N, Bansal RC (2007). Aortic valve replacement improves survival in severe aortic stenosis associated with severe pulmonary hypertension. *Annals of Thoracic Surgery*.

[41] Khandhar S, Varadarajan P, Turk R (2009). Survival benefit of aortic valve replacement in patients with severe aortic regurgitation and pulmonary hypertension. *Annals of Thoracic Surgery*.

[42] Naidoo DP, Mitha AS, Vythilingum S, Chetty S (1991). Pulmonary hypertension in aortic regurgitation: early surgical outcome. *Quarterly Journal of Medicine*.

[43] Shapiro BP, McGoon MD, Redfield MM (2007). Unexplained pulmonary hypertension in elderly patients. *Chest*.

[44] Borlaug BA, Nishimura RA, Sorajja P, Lam CSP, Redfield MM (2010). Exercise hemodynamics enhance diagnosis of early heart failure with preserved ejection fraction. *Circulation: Heart Failure*.

[45] Hunt SA, Abraham WT, Chin MH (2009). 2009 focused update incorporated Into the ACC/AHA 2005 guidelines for the diagnosis and management of heart failure in adults. A report of the American College of Cardiology Foundation/American Heart Association task force on practice guidelines developed in collaboration with the International Society for Heart and Lung Transplantation. *Journal of the American College of Cardiology*.

[46] http://clinicaltrials.gov/ct2/show/NCT00763867?term=relax&rank=00763861.

[47] Guglin M, Khan H (2010). Pulmonary hypertension in heart failure. *Journal of Cardiac Failure*.

[48] Dini FL, Nuti R, Barsotti L, Baldini U, Dell’Anna R, Micheli G (2002). Doppler-derived mitral and pulmonary venous flow variables are predictors of pulmonary hypertension in dilated cardiomyopathy. *Echocardiography*.

[49] Cody RJ, Haas GJ, Binkley PF, Capers Q, Kelley R (1992). Plasma endothelin correlates with the extent of pulmonary hypertension in patients with chronic congestive heart failure. *Circulation*.

[50] Wei CM, Lerman A, Rodeheffer RJ (1994). Endothelin in human congestive heart failure. *Circulation*.

[51] Abramson SV, Burke JF, Kelly JJ (1992). Pulmonary hypertension predicts mortality and morbidity in patients with dilated cardiomyopathy. *Annals of Internal Medicine*.

[52] Cappola TP, Felker GM, Kao WHL, Hare JM, Baughman KL, Kasper EK (2002). Pulmonary hypertension and risk of death in cardiomyopathy: patients with myocarditis are at higher risk. *Circulation*.

[53] Kjaergaard J, Akkan D, Iversen KK (2007). Prognostic importance of pulmonary hypertension in patients with heart failure. *American Journal of Cardiology*.

[54] Delgado JF, Gomez-Sanchez MA, Saenz de la Calzada C (2001). Impact of mild pulmonary hypertension on mortality and pulmonary artery pressure profile after heart transplantation. *Journal of Heart and Lung Transplantation*.

[55] Mehra MR, Kobashigawa J, Starling R (2006). Listing criteria for heart transplantation: International Society for Heart and Lung Transplantation guidelines for the care of cardiac transplant candidates-2006. *Journal of Heart and Lung Transplantation*.

[56] Costard-Jackle A, Fowler MB (1992). Influence of preoperative pulmonary artery pressure on mortality after heart transplantation: testing of potential reversibility of pulmonary hypertension with nitroprusside is useful in defining a high risk group. *Journal of the American College of Cardiology*.

[57] Zakliczynski M, Maruszewski M, Pyka L (2007). Effectiveness and safety of treatment with sildenafil for secondary pulmonary hypertension in heart transplant candidates. *Transplantation Proceedings*.

[58] Martin J, Siegenthaler MP, Friesewinkel O (2004). Implantable left ventricular assist device for treatment of pulmonary hypertension in candidates for orthotopic heart transplantation—a preliminary study. *European Journal of Cardio-Thoracic Surgery*.

[59] Salzberg SP, Lachat ML, von Harbou K, Zünd G, Turina MI (2005). Normalization of high pulmonary vascular resistance with LVAD support in heart transplantation candidates. *European Journal of Cardio-Thoracic Surgery*.

[60] Liden H, Haraldsson A, Ricksten S-E, Kjellman U, Wiklund L (2009). Does pretransplant left ventricular assist device therapy improve results after heart transplantation in patients with elevated pulmonary vascular resistance?. *European Journal of Cardio-Thoracic Surgery*.

[61] Alba AC, Rao V, Ross HJ (2010). Impact of fixed pulmonary hypertension on post-heart transplant outcomes in bridge-to-transplant patients. *Journal of Heart and Lung Transplantation*.

[62] Mikus E, Stepanenko A, Krabatsch T Reversibility of fixed pulmonary hypertension in left ventricular assist device support recipients.

[63] John R, Liao K, Kamdar F, Eckman P, Boyle A, Colvin-Adams M (2010). Effects on pre- and posttransplant pulmonary hemodynamics in patients with continuous-flow left ventricular assist devices. *Journal of Thoracic and Cardiovascular Surgery*.

[64] Califf RM, Adams KF, McKenna WJ (1997). A randomized controlled trial of epoprostenol therapy for severe congestive heart failure: the Flolan International Randomized Survival Trial (FIRST). *American Heart Journal*.

[65] Anand PI, McMurray PJ, Cohn PJN (2004). Long-term effects of darusentan on left-ventricular remodelling and clinical outcomes in the EndothelinA Receptor Antagonist Trial in Heart Failure (EARTH): randomised, double-blind, placebo-controlled trial. *The Lancet*.

[66] Packer M, McMurray J, Massie BM (2005). Clinical effects of endothelin receptor antagonism with bosentan in patients with severe chronic heart failure: results of a pilot study. *Journal of Cardiac Failure*.

[67] Colucci WS, Elkayam U, Horton DP (2000). Intravenous nesiritide, a natriuretic peptide, in the treatment of decompensated congestive heart failure. *The New England Journal of Medicine*.

[68] Mehra MR, Ventura HO, Kapoor C, Stapleton DD, Zimmerman D, Smart FW (1997). Safety and clinical utility of long-term intravenous milrinone in advanced heart failure. *American Journal of Cardiology*.

[69] Lewis GD, Shah R, Shahzad K (2007). Sildenafil improves exercise capacity and quality of life in patients with systolic heart failure and secondary pulmonary hypertension. *Circulation*.

[70] Guazzi M, Samaja M, Arena R, Vicenzi M, Guazzi MD (2007). Long-term use of sildenafil in the therapeutic management of heart failure. *Journal of the American College of Cardiology*.

